# Differential Subcellular Distribution of Cytokinins: How Does Membrane Transport Fit into the Big Picture?

**DOI:** 10.3390/ijms22073428

**Published:** 2021-03-26

**Authors:** Daniel Nedvěd, Petr Hošek, Petr Klíma, Klára Hoyerová

**Affiliations:** 1 The Czech Academy of Sciences, Institute of Experimental Botany, 165 02 Prague, Czech Republic; dan@nedved.eu (D.N.); hosek@ueb.cas.cz (P.H.); 2 Department of Biochemistry, Faculty of Science, Charles University, 128 00 Prague, Czech Republic

**Keywords:** cytokinin transport, membrane transport, ABCG14, PUP14, AZG1, AZG2, cytokinin distribution, cytokinin hydrophobicity

## Abstract

Cytokinins are a class of phytohormones, signalling molecules specific to plants. They act as regulators of diverse physiological processes in complex signalling pathways. It is necessary for plants to continuously regulate cytokinin distribution among different organs, tissues, cells, and compartments. Such regulatory mechanisms include cytokinin biosynthesis, metabolic conversions and degradation, as well as cytokinin membrane transport. In our review, we aim to provide a thorough picture of the latter. We begin by summarizing cytokinin structures and physicochemical properties. Then, we revise the elementary thermodynamic and kinetic aspects of cytokinin membrane transport. Next, we review which membrane-bound carrier proteins and protein families recognize cytokinins as their substrates. Namely, we discuss the families of “equilibrative nucleoside transporters” and “purine permeases”, which translocate diverse purine-related compounds, and proteins AtPUP14, AtABCG14, AtAZG1, and AtAZG2, which are specific to cytokinins. We also address long-distance cytokinin transport. Putting all these pieces together, we finally discuss cytokinin distribution as a net result of these processes, diverse in their physicochemical nature but acting together to promote plant fitness.

## 1. Introduction

Cytokinins (CKs), a group of phytohormones, mediate sensitive and potent regulation of wide variety of growth and developmental processes in plants [1,2]. To keep the CK signal intensity at the desired level, plants need efficient mechanisms to regulate the concentration of active CK forms at specific cellular sites. These mechanisms comprise biochemical reactions (including both formation of biologically active CKs from their precursors and their subsequent metabolic inactivation) and transport processes occurring at biological membranes. Distribution of different enzymes involved in the former varies throughout the subcellular compartments. This fact accounts for compartmentalization of diverse CK species and their enzymatic reactions [3].

Biosynthesis of the most prominent CKs in plants begins with prenylation of adenosine diphosphate (ADP) or triphosphate (ATP). This reaction is catalysed by ADP/ATP isopentenyltransferases (ADP/ATP IPTs) [4,5]. The isoprenoid moiety can be hydroxylated by specific cytochrome P450 (CYP) monooxygenases CYP735A1 and 2 [6]. Such intermediates are converted to free CK bases, which can interact with CK receptors, triggering a cellular response to the CK signal [7]. Chen and Kristopeit isolated purine-specific nucleotidases [8] and nucleosidases, respectively [9]. These findings hint that CK activation could occur via two subsequent reactions. Later, Kurakawa et al. discovered an enzyme family dubbed “Lonely Guy” (LOG), the members of which are able to activate CKs on their own, i.e., with no other enzymes involved in the pathway [10]. Further research suggested that the LOG-mediated CK activation pathway is dominant in plants [11,12].

CKs in the form of free bases can be inactivated either by oxidative degradation or via covalent conjugation with another low-molecular-weight compound, thus forming CK derivatives. The former reaction is catalysed by CK oxidases/dehydrogenases (CKXs). CK degradation yields adenine and an oxidized form of the corresponding side-chain. The oxidative degradation of CKs is irreversible [13,14]. The most prominent CK conjugates are those containing a glucose moiety, i.e., glucosides. Reactions converting free CK bases to glucosides (or glycosylation reactions) are catalysed by UDP glycosyl transferases (UGTs) [15]. While CK *O*-glucosides are readily converted back to free bases by a β-glucosidase [16], *N*-glycosylation is often considered irreversible. However, a recent work published by Hošek et al. suggests otherwise [17]. Apart from CK nucleosides, nucleotides, and glucosides, conjugates with disaccharides or amino acids have been described as well [18].

Biologically active CKs trigger physiological plant responses through a series of transcriptional events mediated via canonical two-component signalling system typically consisting of a sensory histidine kinase (HK) and response regulators (RRs). In *Arabidopsis*, a multi-step phosphorelay system including CK receptors AHK2, AHK3, CRE1/AHK4/WOL1, histidine phosphotransfer proteins (AHP1-5 and AHP6 lacking His residue), and type-A and type-B ARRs was described [2,19].

Indeed, the localization of CK perception system determines the sites of ignition of CK signalling cascade once the active CK is present. However, there is a discrepancy concerning the subcellular localization of the CK receptors as well as HK substrate preferences. Initially, extracellular localization has been proposed for CRE1/AHK4/WOL1 [20,21] and AHK3 [22]. On the other hand, later experiments have shown that fusion constructs of these two AHKs with green fluorescent protein (GFP) reporter reside mainly on the endoplasmic reticulum (ER) membrane [23,24]. The preferential role of ER-localized HKs has been further supported [19,25]. However, recent studies clearly demonstrated that *Arabidopsis* CK receptors localize to plasma membrane as well [26,27], supporting an earlier hypothesis that depletion of CKs from apoplast dampens CK signalling output [28].

Furthermore, it was shown that AHK4 localization depends on the cell type [27]. It might be expected that other, so far unknown factors affect CK receptor distribution too. Considering that HKs substrate preference varies [29] it is hard to connect CK metabolism and transport with direct signalling output.

CK membrane transport consists of several processes with diverse thermodynamic and kinetic aspects, tightly linked to the chemical properties of individual CK species. Recent findings also suggest particular roles of CK-specific membrane-bound carriers during certain developmental events. This review aims to summarize the results of CK transport research so far and to put them within a frame of basic physicochemical principles of membrane transport mechanisms.

## 2. The Structural Variety of Naturally Occurring and Synthetic Cytokinins

**Figure 1:**
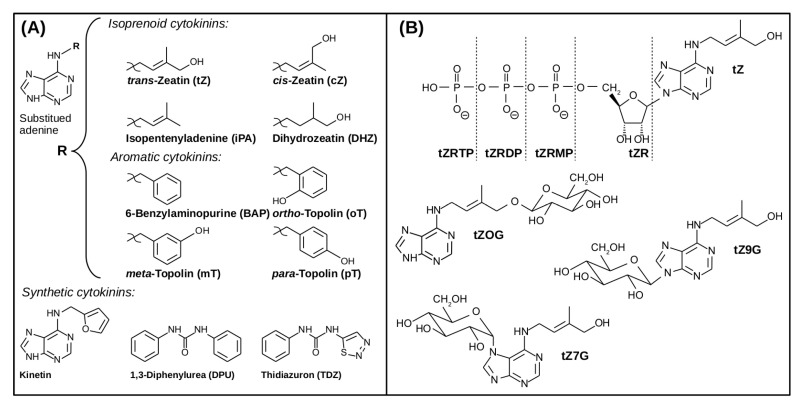
Structures of major cytokinins (CKs) in form of free bases (**A**) and different forms of *trans*-zeatin (**B**). Taken from [32]; tZR: *trans*-zeatin riboside; tZRMP: *trans*-zeatin riboside monophosphate; tZRDP: *trans*-zeatin riboside diphosphate; tZRTP: *trans*-zeatin riboside triphosphate; tZOG: *trans*-zeatin *O*-glucoside; tZ7G: *trans*-zeatin *N^7^*-glucoside; tZ9G: *trans*-zeatin *N^9^*-glucoside. Remaining abbreviations are explained in the figure.

Various mechanisms of CK transport closely tie to their structural and functional diversity. Naturally occurring CKs can be characterized as derivatives of adenine. Their molecules bear a side-chain moiety bound to the N6 atom (i.e., the primary amine group of adenine). The composition and structure of the said side-chain play a pivotal role in chemical diversity among major CKs (Figure 1). Regarding the general chemical character of their side-chains, naturally occurring CKs can be further classified as isoprenoid or aromatic [30,31].

Isoprenoid CKs’ side-chain derives from a dimethylallyl moiety—a five-carbon atomic group bearing a double bond. Dimethylallyl pyrophosphate is a common precursor of isoprenoid CKs and secondary plant metabolites [33]. Isopentenyladenine (iP) contains a dimethylallyl side-chain with no further modifications. Terminal hydroxylation of the dimethylallyl side-chain yields zeatins. Due to the stereochemistry of double bonds, two zeatin isomers exist—*cis*-zeatin (cZ) and *trans*-zeatin (tZ). Both these isomers occur in plants. The relative amounts of cZ-type and tZ-type CKs vary throughout different plant species and during different developmental stages within a plant’s lifetime [34]. Despite their structural similarity, CK-specific enzymes and receptors can discriminate between the two zeatin isomers [35].

Bassil et al. isolated an enzyme supposed to catalyse the *cis*-*trans* isomerization of zeatin [36]. However, a later study published by Hluska et al. challenged this view. The authors showed that no significant change of cZ to tZ ratio occurred in *Arabidopsis* plants expressing the said enzyme. They suggested that the putative cis-trans isomerase actually catalysed hydrolysis of flavin adenine dinucleotide (FAD) to flavin adenine mononucleotide (FMN). FMN could be then readily excited by light and induce non-enzymatic photoisomerization of zeatin [37].

Gajdošová et al. studied the *cis*-*trans* isomerization as a potential source of artefacts in CK profiling. They have concluded that such a reaction in plant samples requires either photochemical or thermal catalysis, due to its relatively high activation energy [34].

Until now the only source of cZ seems to be degraded tRNA as shown by Miyawaki et al. Using *ipt2ipt9* double mutant they demonstrated complete lack of cZ-type metabolites in the mutant pointing at tRNA IPT as a key enzyme in cZ biosynthesis [38].

Another known type of isoprenoid CKs is dihydrozeatin (DHZ). Its side-chain is hydroxylated, similarly to cZ or tZ, and fully saturated. Martin et al. purified a putative zeatin reductase, supposed to produce DHZ by hydrogenation of zeatins [39]. The authors observed DHZ formation in a mixture containing the isolated enzyme, radiolabelled tZ, and nicotinamide adenine dinucleotide phosphate (NADPH). They saw no such reaction with cZ, tZR, *O*-xylosyl zeatin or iP as substrates. Later, NADPH-dependent conversion of cZ to DHZ was shown in pea leaf [40]. Recently, a correlation analysis of CK metabolites in potato suggested there might be interconversions between DHZ riboside and tZR with possible involvement of cZ-type CKs [41].

The side-chains of naturally occurring aromatic CKs are based on a benzyl moiety. The simplest aromatic CK, whose side-chain is not further modified, is called 6-benzylaminopurine (BAP). BAP can be both prepared synthetically and isolated from *Arabidopsis* and poplar [31,42]. Formal monohydroxylation of the aromatic ring at the side-chain of BAP yields topolins. The monohydroxylation can occur at three sites, yielding three possible topolin isomers, called *ortho*- (oT), *meta*- (mT), and *para*-topolin (pT). All three topolins, as well as their metabolites, have been found in plants [31,43]. Methyl ethers of oT (*ortho*-methoxytopolin—MeoT), mT (*meta*-methoxytopolin—MemT), and their respective nucleosides were identified too [42]. Another class of aromatic CKs, 6-(3-methylpyrrol-1-yl)purine (MPP) and its derivatives, has been identified as a metabolic product of tZ. Formation of MPP has been observed in maize [44] as well as in cereal pathogen *Fusarium pseudograminearum* [45]. Interestingly, chemical synthesis of MPP from tZR had been already carried out years ago [46].

As pointed out by Hluska et al., the current knowledge of aromatic CK metabolism is sparse [29]. Still, there have been few reports on aromatic CK glycosylation; BAP has been *N*-glycosylated by UGT76C1 and 2 [15] and topolins have been *O*-glycosylated by recombinant *O*-glucosyl transferases [47]. Oxidative cleavage of pT by several AtCKXs has been observed too [48]. Biosynthesis of aromatic CKs remains unknown, with the exception of MPP and its derivatives [44,45].

Some other naturally occurring CKs are synthesized not by plants themselves, but rather by plant pathogens. These include various methylated, thiomethylated, and deoxyribosylated variants of iP and zeatin-type CKs [49,50,51,52]. These compounds have been shown to mimic physiological CK activity, effectively disrupting the hormonal balance in host plants and facilitating the infection.

Kinetin is an example of a synthetic CK. It is a derivative of adenine, like its naturally occurring counterparts. Its side-chain consists of an aromatic furan moiety linked to the N6 amino group via a methylene bridge. Another type of synthetic CKs comprises derivatives of phenylurea, such as diphenylurea (DPU), *N*-(2-chloro-4-pyridyl)-*N*′-phenylurea (CPPU), thidiazuron (TDZ), and its monohydroxylated derivatives [5,31,47]. Chemical synthesis is also employed to produce novel CK derivatives [18,53].

## 3. Cytokinin Structures Affect Their Physical and Chemical Properties

Structures of CKs govern both their physicochemical and biological properties (such as ability to trigger cellular response via CK signalling system). Different properties of naturally occurring free CK bases can be accounted to the differences in their side-chain compositions. Similarly, metabolites or precursors of a given free base vary in their properties due to different moieties present in their molecules.

**Figure 2:**
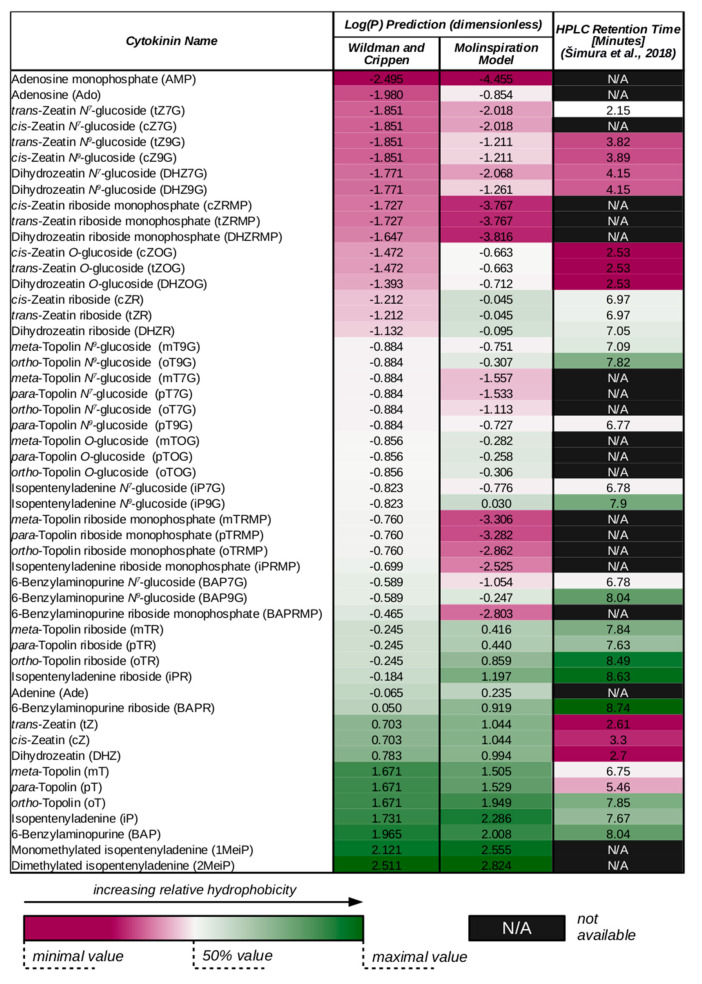
Hydrophobicity of various cytokinin species (CK) has been predicted using cheminformatics tools. In the second column, log(*P*) values are estimated according to [54]; this method yields log(*P*) as a sum of individual Wildman-Crippen atomic contributions. Calculation of Wildman-Crippen contributions has been done using RDKit: Open-source cheminformatics software (http://www.rdkit.org, accessed on 4 January 2021). In the third column, log(*P*) values are estimated using a method provided by Molinspiration Cheminformatics free web services (https://www.molinspiration.com, accessed on 4 January 2021). To compare these predictions with actual experimental data, retention times of internal standards for high-performance liquid chromatography (HPLC) separation of CKs taken from [55] are given in the last column. For all three datasets, relative hydrophobicity range is visualized using the colour scale given below the table. The colour range is normalized within each column. Note that not all of the listed CKs were analysed in [55], as indicated by missing values (“N/A”).

**Figure 3:**
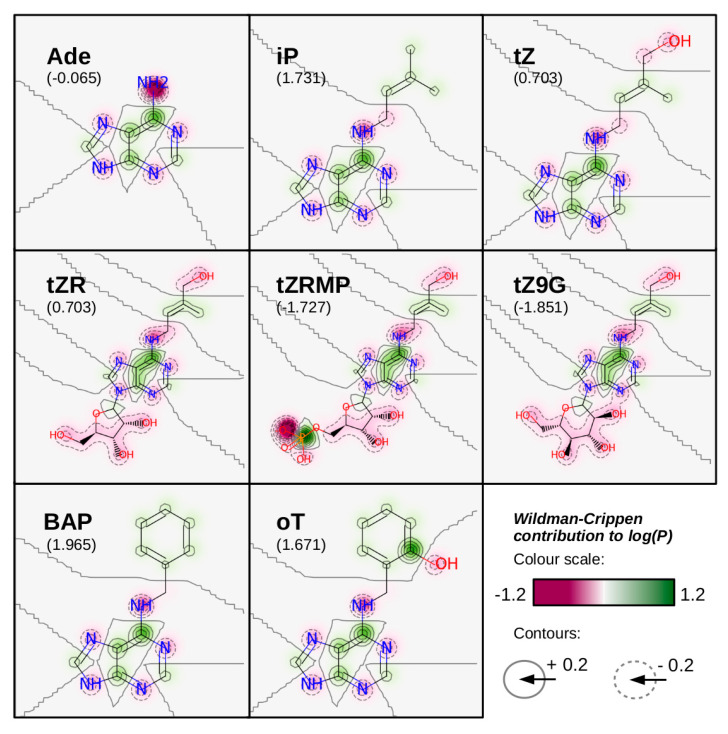
For selected CKs, Wildman-Crippen contributions of individual atoms to the value of log(*P*) have been predicted according to [54]. Gaussian distribution of the contributions have been visualized and juxtaposed with the corresponding molecular structures. Negative contributions (which decrease the overall hydrophobicity of the molecule) are depicted in shades of pink, while positive contributions (which increase the overall hydrophobicity) are depicted in shades of green (refer to the colour-bar in the bottom right corner of the figure). Increments of Wildman-Crippen contribution levels are also expressed by solid (above zero levels) and dashed contours (below zero levels). For each molecule, the Wildman-Crippen value of log(*P*) is given in the parentheses. Note that contributions of hydrogen atoms, which have been included in predicting total log(*P*) values, are not shown. Calculation and visualization of the Wildman-Crippen contributions have been performed using RDKit: Open-source cheminformatics software (http://www.rdkit.org, accessed on 4 January 2021). Ade: adenine; iP: isopentenyl adenine; tZ: *trans*-zeatin; tZR: *trans*-zeatin riboside; tZRMP: *trans*-zeatin riboside monophosphate; tZ9G: *trans*-zeatin *N^9^*-glucoside; BAP: 6-benzylaminopurine; oT: *ortho*-topolin; *P*—octanol-water partition coefficient.

One of the important chemical features of CKs to be discussed is their hydrophobicity. This property can be characterized by octanol-water partition coefficient, *P*, or its logarithm. Wildman and Crippen published a method to predict log(*P*) of a given molecule by summing up individual contributions of all its atoms [54]. The value of each contribution depends both on the atom type and its surroundings. To demonstrate this prediction method, log(*P*) values of various CKs have been estimated and listed in Figure 2 (in comparison with another prediction method and retention times of internal HPLC CK standards provided by Šimura et al. in [55]). Additionally, individual atomic contributions to the net log(*P*) values of selected CKs, evaluated according to Wildman and Crippen [54], are depicted in Figure 3.

Molecular hydrophobicity governs, for instance, the kinetics of diffusion across biological membranes, which is one of the basic mechanisms of cellular transport. Furthermore, hydrophobic interactions play their part in interactions between low-molecular substances (such as CKs) and proteins (including CK-specific receptors, transporters, and enzymes) [56]. Naturally occurring CKs possess relatively hydrophobic side-chains, which has proved useful for CK isolation from plant material by solid-phase extraction (SPE) and their further analysis by HPLC [55,57,58,59].

Several observations can be made considering Figure 2 data. For instance, while both prediction methods agree that hydrophobicities of tZ and cZ should be equal, the HPLC data show that tZ is eluted slightly earlier, suggesting that other molecular properties, such as double bond stereochemistry, could also play their roles in the interaction between analytes and HPLC columns. However, this effect is observed only for free zeatin bases and not the other forms. One can also notice that while Wildman-Crippen method predicts all free topolin bases to be of equal hydrophobicity, the Molinspiration model considers oT slightly more hydrophobic than the other two, which better corresponds to the HPLC data.

Both predictions generally consider CK glucosides less hydrophobic than the corresponding ribosides and the ribosides less hydrophobic than the corresponding free bases. This pattern suggests that discrimination among free CK bases, ribosides or glucosides observed for some membrane-bound transporters [28,60,61,62], enzymes [15,48] or CK receptors [63,64,65] could be at least partly caused by the differences in substrate hydrophobicity. Interestingly, in the HPLC data, zeatin-type ribosides are shown to be more hydrophobic than both free bases and glucosides.

Aromatic and iP-type CKs are shown to be more hydrophobic than their zeatin-type counterparts in both predictions and HPLC data. Following the idea presented in the previous paragraph, this hydrophobicity differences might be an explanation for different apparent affinities towards zeatin-type and iP-type substrates observed for some membrane-bound transporters [61,66,67,68], enzymes [15,48] or CK receptors [47,64].

CKs can also act as acids or bases, undergoing deprotonation or protonation in various pH. The N6 amino group, which binds the specific side-chains, is mostly protonated at pH < 3, bearing a positive charge. The N9 imine group can be deprotonated at pH > 11, becoming negatively charged. Similarly, the 2′-hydroxyl groups present on ribosyl moieties in CK nucleosides and nucleotides become mostly deprotonated at pH > 12. The phosphate group of CK nucleotides is neutral at pH < 1, once deprotonated at pH < 6, and twice deprotonated at pH < 12 [69].

Most of these proton transfer reactions occur at non-physiological pH values, which makes them irrelevant to CK transport processes. Still, CK nucleotides remain negatively charged at neutral pH, which effectively disables their diffusion across biological membranes under physiological conditions.

## 4. Cytokinin Content Varies in Different Subcellular Compartments

Differential distribution of CKs affects their transport, as both its thermodynamics and kinetics depend on CK concentration in various cellular compartments, as well as in the apoplast. Conversely, knowledge of CK subcellular localization can help to decide whether CK transport is expected to be involved in specific situations as a regulatory process. Important findings in this field, as well as the methodology of CK subcellular fractionation are reviewed in [3].

A comprehensive study of CK distribution in *Arabidopsis* (a dicot) and barley (a monocot) leaf cells was carried out by Jiskrová et al. [70]. In *Arabidopsis*, tZ was mostly extracellular, with a small fraction localized in cytoplasm, similarly to tZR and tZ-type glucosides; tZRMP was found in the cytoplasm as well. iP and iPR were predominantly localized in the cytoplasm, while most of iPRMP was found in the extracellular space. The iP-type glucosides were found in both the extracellular space and vacuoles. cZ was extracellular, cZR intracellular, cZRMP was found in both extracellular space and cytoplasm, and cZ-type glucosides were localized in the extracellular space and vacuoles. Of DHZ-type CKs, only DHZ7G was detected in a significant amount; it was localized in the extracellular space and vacuoles. In barley, apoplast was dominated by tZ9G and cZOG with a noticeable presence of the active bases iP and tZ as well as iPRMP. In contrast to *Arabidopsis*, vacuolar content was enriched in iP and cytoplasmic content in iPR. cZ forms made up for over 60% of both the cytoplasmic and the vacuolar cytokinins content in barley [70].

The extracellular localization of tZR reported by Jiskrová et al. [70] is in agreement with results of CK profiling in xylem sap, where tZR was the major CK species [67,71,72,73]. In phloem sap, iP-type nucleosides and nucleotides were the dominant species [67,74]. A notable spatial discrepancy appears to exist between the reported extracellular localization of CK glucosides [70] and the described cytoplasmic localization of CK-specific glycosyl transferases [75,76].

In chloroplasts from tobacco and wheat, a wide range of CKs has been detected. The results of CK profiling varied in samples collected at the end of light and dark periods, both in the total CK amount and in the composition of the CK fraction. Notably, dark-treated chloroplasts contained much larger amounts of CK glucosides, compared to their light-treated counterparts [77].

Another CK content analysis has been carried out in *Arabidopsis* root cell protoplast, as well as in apoplastic and symplastic fractions. Interestingly, CK glucosides have been found in high abundance in both symplastic fraction and protoplast. On the other hand, free bases and ribosides were more or less equally abundant in both symplastic and apoplastic fractions [26].

Until now endogenous cytokinin compartmentalization is still far from being unified. The development of more sensitive analytical methods as well as cell and organelle sorting [78] should lead to more precise determination of minute amounts of CK derivatives within the cell compartments.

## 5. Cytokinin Transport at the Cellular Level Occurs via Thermodynamically and Kinetically Diverse Processes

Compounds moving between apoplast and cytoplasm, two cells, or different compartments at the subcellular level have to cross biological membranes. These membranes consist of an amphiphilic lipid bilayer and many proteins varying in size, shape, and function.

The means of transport across the biological membrane comprise simple diffusion, facilitated diffusion, primary, and secondary active transport. In this part, we summarize these processes, their basic mathematical description, and discuss them in terms of CK cellular transport.

From the thermodynamic point of view, membrane transport processes can be classified as either passive, if they can occur spontaneously, or active, if they require an external energy source. At a constant temperature and pressure, the energy balance of a transport process can be quantified using Gibb’s free energy, Δ*G*. For a transport process between two compartments, Δ*G* can be expressed as:

(1) $$\Delta G=RTln\frac{c_{t}}{c_{s}}+zFV$$

Here, *R* is the universal gas constant (approx. 8.3145 J × K^−1^ × mol^−1^), *T* is thermodynamic temperature, *c_s_* and *c_t_* are concentrations of the transported compound in the source and the target compartment, respectively, *z* is the charge number of the compound, *F* is the Faraday constant (approx. 96,485 C × mol^−1^), and *V* is the voltage between the two compartments. Translocation processes characterized by negative Δ*G* can occur via passive routes, whereas those characterized with positive Δ*G* are realized by means of active transport. The latter utilizes energy provided by an exergonic process. According to the type of this process, active transport is further classified as primary or secondary.

The primary active transport is directly coupled to the hydrolysis of adenosine triphosphate (ATP). Both the hydrolysis and the translocation are catalysed by multi-domain membrane carriers belonging to the “ATP-Binding Cassette” (ABC) family. A typical ABC protein is composed of two nucleotide-binding domains (NBDs) and two transmembrane domains (TMDs). Some ABCs are expressed as either homodimers or heterodimers, with each protomer containing one NBD and one TMD [79]. In plants, ABCs are further divided into eight subfamilies (ABCA—ABCG and ABCI). They recognize a wide range of substrates, including some phytohormones [80].

The secondary active transport employed by most members of plant ENT and PUP families utilizes energy gained from passive translocation of another substrate [81]. Both substrates are transported by the same carrier. In plants, the secondary active transport is often linked to the proton gradient, which can be also expressed as a difference of pH between two compartments.

While the thermodynamics help us to discuss the energy balance, it does not tell us how fast the transport processes occur. To access this kind of information, we need kinetic equations describing the transport flux and/or the resulting temporal changes in the concentrations of the transported substances. Passive transport occurs via simple or facilitated diffusion. The latter is mediated by membrane-bound carriers, which results in different kinetic descriptions of the two diffusion types. Active transport always requires the membrane carries, and therefore it shares its kinetic characteristics with facilitated diffusion. However, the thermodynamic difference remains—facilitated diffusion, a passive process, cannot occur if Δ*G* is positive.

The kinetics of simple diffusion is described by classical Fick’s laws [82]. The first law describes the relationship between the diffusion flux, $\vec{J}$, and the concentration gradient, ∇*c*. It states that:

(2) $$\vec{J}=-D\nabla c$$

where *D* is the mass diffusivity, a parameter depending on both environment and the properties of the transported substance. Note that *∇* (pronounced “nabla”) is a vector of partial derivatives with respect to all three spatial coordinates, denoted as x, y, and z:

(3) $$\nabla =\left(\frac{\partial }{\partial x},\frac{\partial }{\partial y},\frac{\partial }{\partial z}\right)$$

In other words, the diffusion flux is proportional to the concentration gradient and it occurs in the opposite direction. Considering a system, where *c* changes only due to diffusion, Fick’s first law can be used to derive the temporal change of concentration as:

(4) $$\frac{\partial c}{\partial t}=D\nabla^{2}c$$

where *t* is the time. This is a formulation of Fick’s second law. The square of *∇*, or the Laplace operator, can be expressed as:

(5) $$\nabla^{2}=\frac{\partial^{2}}{\partial x^{2}}+\frac{\partial^{2}}{\partial y^{2}}+\frac{\partial^{2}}{\partial z^{2}}$$

Models based on simple diffusion and Fick’s laws have been used to describe short-distance apoplastic movements and membrane transport of various plant hormones, including CKs [83,84,85]. Simple diffusion across biological membranes is generally allowed to small, hydrophobic, and non-charged molecules. In Figure 2, hydrophobicity of some CKs is predicted in terms of log(*P*), which the molecular affinity towards hydrophobic and hydrophilic environments. CKs able to readily diffuse through the biological membrane should have relatively high log(*P*) to be able to pass through its lipid core. Considering both Figure 2 predictions, log(*P*) of free CK bases ranges from approximately 0.7 to 2.0; in other words, their affinities to hydrophobic environments are predicted to be 5 to 100 times higher in comparison with the hydrophilic ones. Therefore, it is worth considering that free CK bases are able to cross biological membranes, unless proved otherwise. Other data from Figure 2 (both Molinspiration prediction and HPLC internal standard elution times) suggest that simple diffusion might be relevant for topolin ribosides as well.

As discussed by Radhika et al., plant pathogen *Rhodococcus fascians* produces mono- and dimethylated derivatives of iP which are fairly hydrophobic, and therefore easily diffuse through membranes, facilitating the bacterial infection [51]. In Figure 2, both these derivatives are predicted to be more hydrophobic than the free CK bases produced by plants.

Weak acids and bases may cross the membrane only non-dissociated. The dissociation equilibrium of a weak acid or base is characterized by a certain value of *pK_A_*. The ratio between concentrations of the dissociated, *c*(*A*^−^), and non-dissociated form of the given compound, *c*(*HA*), can be written using one of the Henderson-Hasselbalch equations:

(6) $$log\frac{c\left(A^{-}\right)}{c\left(HA\right)}=pH-pK_{A}$$

While this equilibrium does not play a significant role in regulating CK transport, it is crucial for the polar distribution of auxins, weakly acidic phytohormones. As reviewed by Zažímalová et al. [86], a significant portion of auxin molecules is protonated in the apoplast, due to its mildly acidic pH (5.5). In such form, it readily diffuses to the cytoplasm, which is rather neutral (7.0). There, the transported molecules deprotonate, becoming negatively charged and therefore trapped within the cells.

Kramer modelled the apoplastic diffusion of several weakly acidic phytohormones: auxin, abscisic acid, and gibberellins [87]. In this model, the weak acid travels through the apoplast in one direction from the transmitter cell to the receiver one. A portion of the protonated acid molecules is continuously trapped by surrounding sink cells. Each weak acid can be characterized by its decay length, *L_apo_*, indicating how far it can travel before its apoplastic concentration decreases to 10% of its initial value. The decay length is defined as:

(7) $$L_{apo}\approx 1.63\frac{\sqrt{Dh}}{P_{eff}}$$

where *h* is the width of the apoplastic route, and *P_eff_* is effective permeability of the sink cell membranes, depending on the dissociation constant of the given weak acid. The dissociation constant determines the rate of constant depletion of the travelling weak acid molecules from the apoplast, which is required in the model. In contrast to the acids, CK dissociation is not a relevant source of this behaviour and CK diffusion from the cytoplasm back to apoplast cannot be ruled out. However, a similar model could be derived, based, for example, on continuous cleavage of CK molecules by apoplastic CKXs [48]. CK degradation rate could be therefore implemented in a similar fashion, with consideration of CKX apoplastic distribution.

Facilitated diffusion is mediated by membrane-bound carriers, such as AtENT7 in the case of CK transport [81,88]. The thermodynamics remains the same as in the case of the simple diffusion (i.e., passive transport). However, a different kind of kinetic model is required to characterize this process. Carrier-mediated transport, including both facilitated diffusion and active transport, can be described using Michaelis-Menten kinetics [89] (English translation and additional commentary provided by Johnson and Goody in [90]). This model expresses the relationship between the transport rate, *v*, and the concentration of the transported compound, or the substrate, as:

(8) $$v=\frac{V_{max}c}{K_{M}+c}$$

where *V_max_* is the limit rate (achieved at complete saturation of the given membrane carrier) and *K_M_* is the substrate concentration at which the transport rate is equal to half *V_max_* (*K_M_* therefore characterize the affinity of the carrier toward the substrate). In the presence of a competitive inhibitor, which binds to the same site of the carrier as the substrate does, the kinetic equation expands into:

(9) $$v=V_{max}\frac{c}{K_{M}+\frac{K_{M}}{K_{I}}c_{i}+c}$$

where *c_i_* is the inhibitor concentration and *K_I_* is the dissociation constant of inhibitor-enzyme complex.

Carrier-mediated transport can be approximated with first-order kinetics if the substrate concentration is relatively low, simplifying Michaelis-Menten equation as follows:

(10) $$c\ll K_{M}\Rightarrow K_{M}+c\approx K_{M}$$

(11) $$v\approx \frac{V_{max}}{K_{M}}c$$

The ratio of $\frac{V_{max}}{K_{M}}$ is equivalent to the first-order rate constant and it can be also interpreted as membrane permeability. More complex models of hormonal homeostasis may express membrane transport with first-order kinetics, considering the requirement of low substrate concentration fulfilled [83,84,87].

On the other side of the concentration range, i.e., when the substrate concentration permanently remains at levels corresponding to practical saturation of the carrier, the transport kinetics become independent of it. In such situation, the Michaelis-Menten equation simplifies to:

(12) $$c\gg K_{M}\Rightarrow K_{M}+c\approx c$$

(13) $$v\approx V_{max}$$

This is an example of zero-order kinetics.

## 6. Equilibrative Nucleoside Transporters Mediate Proton-Dependent Transport of Cytokinin Ribosides

CKs (and some other derivatives of adenine) are recognized as non-specific substrates by some membrane-bound transporters of nitrogenous bases and nucleosides. One family of such carriers is called the “Equilibrative Nucleoside Transporters” (ENTs). As the name suggests, ENTs recognize various nucleosides [81]. ENTs are present within genomes of most eukaryotes. In plants, they were identified for the first time thanks to the homology with their human counterparts [81,91,92].

In the *Arabidopsis* genome, eight *ENT* genes have been predicted. Five of them (*AtENT1*, *3*, *4*, *6*, and *7*) have been characterized in terms of their products’ substrate specificities and transport mechanisms [62,67,81,88,91,92,93,94,95,96]. In addition, three function products of *ENT* genes found in rice (*Oryza sativa*) were biochemically characterized [66].

Most plant ENTs mediate secondary active transport coupled to the proton gradient [81]. This fact was confirmed in several studies, in which dependence of the ENT activity on pH was studied [88,94,96]. In other studies, effects of protonophores, which erase the proton gradient and disable proton-dependent membrane transport processes were examined [88,92]. Examples of such protonophores include carbonyl cyanide *m*-chlorophenyl hydrazone (CCCP) or 2,4-dinitrophenol (DNP). However, AtENT7 did not respond to either a change of pH or application of a protonophore [88,94]. Therefore, ENT7 likely mediates facilitated diffusion, rather than secondary active transport, remaining true to the “equilibrative” part of its name. Analogously, the best-characterized human ENTs, hENT1 and 2, mediate facilitated diffusion as well. However, two other hENTs are suggested to mediate secondary active transport, possibly coupled to the proton gradient [97,98].

AtENT1 was the first identified plant ENT. It was shown to recognize common nucleosides, except for uridine, while the corresponding free bases were not recognized as substrates at all [91,92]. AtENT1 localizes to both plasma membrane and tonoplast, mediating the release of nucleosides and RNA breakdown products from vacuole to cytoplasm. Therefore, AtENT1 could contribute to the CK homeostasis by providing adenosine for cytosolic CK biosynthetic pathways [93]. However, *atent1* mutation did not produce any significant change of either CK response or CK uptake by *Arabidopsis* hypocotyl explants [62].

In contrast, the *atent3* loss-of-function mutation led to a decrease in the accumulation of tZR and iPR in *Arabidopsis* hypocotyl explants. The accumulation of the corresponding free bases remained unchanged [62]. In another experiment, *AtENT3* was expressed in yeast cells accumulating adenosine. Upon addition of either tZR or iPR, only a weak decrease of adenosine uptake was observed [67]. Similar results were obtained for AtENT7. On the contrary, in yeast cells expressing *AtENT6*, significant inhibition of adenosine uptake by iPR was observed, while inhibition by tZR was comparable to that in yeast cells expressing *AtENT3* and *7* [67]. The authors suggest that AtENT6 may be involved in tZR and iPR compartmentalization. Both AtENT3 and 6 are localized to plasma membrane [88,95].

AtENT8 was originally identified as a suppressor of AtIPT8. Subsequently, it was found that the *atent8* loss-of-function mutation reduces plants’ sensitivity towards exogenously supplied CK ribosides, while the sensitivity towards the corresponding free bases remains unchanged. Uptake of iPR by *atent8* hypocotyl explants also decreased (although tZR uptake was not altered). Conversely, upon over-expression of *AtENT8*, sensitivity of plants towards exogenous CK ribosides (but not free bases) was increased [62].

Expression patterns of *AtENTs* in five organs (root, stem, leaf, flower, and silique) were examined by [95]. The expression pattern of *AtENT8* throughout various developmental stages was later studied in greater detail [62]. The expression of *AtENT6* was further found to be confined to the vasculature, suggesting a possible involvement of AtENT6 in long-distance nucleoside transport [67]. Expression patterns of ENTs relevant to CK transport are summarized in Table S1.

OsENT2 from rice was implied to act as a CK transporter as well. When expressed in yeast cells accumulating adenosine, a drop in adenosine uptake was observed in presence of iPR (but not in presence of tZR). Similarly to most AtENTs, OsENT2-mediated adenosine uptake depends on pH and is partially hindered by CCCP. Direct uptake of tZR and iPR in transgenic yeast cells was also observed. The affinity of OsENT2 towards tZR was significantly lower than towards iPR. *OsENT2* is expressed mainly in roots and to a lesser extent in stems and leaf sheaths [66].

## 7. Purine Permeases Are Involved in Proton-Dependent Transport of Free Cytokinin Bases

Another protein family contributing to the CK membrane transport is that of “Purine Permeases” (PUPs). Most PUPs recognizing CKs are non-specific, similarly to the ENTs discussed above. However, unlike ENTs, PUPs recognize their substrates in the form of free bases, as reviewed in [81]. Hildreth et al. used bioinformatics search tools to predict PUPs to be only found in vascular plants [99]. In the *Arabidopsis* genome, 23 *PUP* genes have been found, though only a few of them have been isolated and biochemically characterized [28,60,61,81,100,101,102]. Thirteen other PUPs have been found in rice [68] and two in tobacco (*Nicotiana tabacum*) [99].

The first discovered PUP was AtPUP1. It was identified via complementation of a yeast mutant deficient in adenine uptake. The AtPUP1-mediated uptake of adenine was hindered upon addition of cytosine, hypoxanthine, nicotine, caffeine, and also two CKs—kinetin and tZ. Corresponding nucleosides and nucleotides were not recognized as AtPUP1 substrates. The inhibition of adenine uptake by both CKs was shown to be competitive. The adenine and cytosine uptakes were dependent on pH and sensitive to the use of protonophores [60]. Based on its function and expression pattern (summarized in Table S1), the authors suggest that the role of AtPUP1 is to import various substrates, such as nucleobases or CKs, from xylem to shoot tissues. Bürkle et al. conducted direct measurements of tZ uptake in yeast cells expressing *AtPUP1*, confirming that AtPUP1 acts as a CK transporter. Furthermore, they found that iP is another substrate of AtPUP1 [61]. Szydlowski et al. showed that AtPUP1 is also involved in the uptake of pyridoxine (vitamin B6), which can be inhibited, among others, by tZ. They also traced AtPUP1 subcellular localization to the plasma membrane [103].

Similar experiments were carried out on AtPUP2. When expressed in yeast cells, *AtPUP2* mediated proton-dependent adenine uptake inhibited by iP, kinetin, BAP, and to a lesser extent by tZ and cZ. The adenine uptake obeyed Michaelis-Menten kinetics, but its rate was significantly lower than upon expression of *AtPUP1*. Expression of *AtPUP2* did not complement mutant yeast deficient in adenine uptake. In contrast, AtPUP3 did not display any transport activity at all [61].

Three PUPs have been suggested to be involved in CK transport in rice: OsPUP1, 4, and 7. OsPUP1 and 7 localize to ER and OsPUP4 to plasma membrane [68,104,105].

*OsPUP1* overexpression in rice plants led to a decrease of tZ, tZR, iP, and DHZ concentrations in shoots; while tZR concentration decreased in roots as well, those of cZ, cZR, and DHZR increased. In panicles, iP, iPR, and cZR concentrations decreased, while those DHZ and DHZR increased. These findings hinted that *OsPUP1* overexpression impairs root-to-shoot CK translocation, which was further confirmed by showing that treating overexpressor roots with endogenous CKs triggers much less pronounced response in shoots that in the wild type. Interestingly, *ospup1* mutants exhibited no significant differences from the wild type, suggesting redundancy among the *OsPUP* genes [105].

*OsPUP4* was identified as a gene responsible for *bg3-D* (“big grain”) phenotype. As the name implies, the phenotype includes larger grains but also taller shoots, shorter roots, and longer leaves in mature plants. The *bg3-D* phenotype corresponded to that caused by *OsPUP4* overexpression. *OsPUP4* expression was reduced by exogenous application of BAP, iP, tZ, and cZ, hinting that the gene is involved in modulating response to CK signal. CK profiling in *bg3-D* plants revealed that iP content decreased in shoots but increased in roots, while cZ and tZ increased in both parts of the plant, suggesting that OsPUP4 is involved in shoot-to-root translocation of iP. This was further confirmed by showing that *bg3-D* plants are more sensitive to exogenous application of CKs to shoots than wild type [104].

The *ospup7* mutant displayed several phenotype alterations, which were explained as results of CK transport impairment and accumulation of CKs in their source organs. Higher amounts of iP and iPR were found in the mutant plants, while the content of tZ-type CKs remained the same as in the wild type. Expression of *OsPUP7* in yeast led to phenotype rescue of mutants deficient in caffeine uptake [68].

In tobacco, PUP-like transporters NUP1 and 2 (“Nicotine Uptake Permease”) were identified thanks to their similarity to AtPUP1. NUP1 was expressed in yeast cells and characterized as a nicotine-specific transporter. Addition of kinetin did not inhibit the nicotine uptake. NUP1 was found to be expressed mostly in root tips and localized to the plasma membrane. While its sensitivity to protonophores was not studied, it was assumed that NUP1 mediates secondary active transport coupled to the proton gradient, based on its homology with AtPUP1 [99].

## 8. Recent Findings Suggest That Proteins AtPUP14, AtABCG14, AtAZG1, and AtAZG2 Are Cytokinin-Specific Transporters with an Important Role in Cytokinin Signalization

All the transporters discussed so far have been non-specific to CKs, meaning that they recognize a wider range of substrates and their exact involvement in CK-mediated processes remains unclear. However, the transport activities of four proteins—AtPUP14, AtABCG14, AtAZG1, and AtAZG2—are linked to distinct features of CK physiology. This fact suggests that CK transport and distribution is an important part of the complex hormonal network in plants.

The role of AtPUP14 was identified by Zürcher et al. [28]. They used CK-specific reporter *TCSn::GFP* (Two Component Signalling Sensor new::Green Fluorescent Protein) developed earlier [106] to find out that prospective cotyledons in *Arabidopsis* embryos did not respond to CK signal, even though CK receptors were actively expressed there. These results led to a hypothesis that members of the PUP family may be involved in precise CK distribution, thus regulating the CK signalization by limiting the availability of biologically active CKs at specific sites. AtPUP14 turned out to be the aptest candidate for such task, given its ubiquitous expression [28,100].

Introduction of AtPUP14-targeting artificial microRNA (*amiRPUP14*) caused ectopic CK signalization in *Arabidopsis* plants, which manifested in several phenotype alterations, such as lateral root suppression or increase in shoot branching. Conversely, inducible expression of *AtPUP14* in *Arabidopsis* embryos reduced endogenous CK response and promoted morphological defects in embryo roots. Visualizing *AtPUP14* and *TSCn::GFP* expression produced complementary patterns. All these results suggest that AtPUP14 down-regulates CK signalling [28].

Uptake assays in mesophyll protoplasts and tobacco microsomes were used to study the biochemical properties of AtPUP14. Transient expression of *AtPUP14* led to an increase in tZ uptake and its rate turned out to be dependent on ATP. Contrarily, tZ uptake in *amiRPUP14* seedlings was hindered. AtPUP14-mediated tZ uptake was inhibited by iP, BAP, and adenine, but not by tZR. Further experiments showed that AtPUP14 is localized to the plasma membrane [28].

The role of another CK-specific transporter, AtABCG14, was reported by two groups within one year. Ko et al. identified AtABCG14 as a transporter potentially involved in root-to-shoot communication via CK transport, given its co-localization with IPTs. Several experiments conducted on *atabcg14* mutants confirmed this hypothesis. Mutant plants displayed growth retardation, which has been rescued by applying exogenous tZ. Mutant shoots also contained less tZ-type CKs than those of the wild type. Grafting *atabcg14* shoots on wild type roots rescued their phenotype while grafting wild type shoots on *atabcg14* roots had no effect. All these results suggest that AtABCG14 is involved in root-to-shoot CK translocation. The authors also tried to study direct CK uptake in yeast cells expressing *AtABCG14*, but they could not detect any transport activity. They hypothesized that the reason for the negative result could be a heterodimeric quaternary structure of biologically active AtABCG14 [107]. As reviewed by Kang et al., some ABC transporters are indeed composed of two different protomers [80]. Le Hir et al. had also shown that AtABCG14 forms heterodimers with AtABCG11, rather than homodimers [108]. However, Ko et al. pointed out that the expression of *AtABCG11* in roots is low, which makes the involvement of said heterodimer in CK shoot-to-root transport unlikely. They tried to identify putative AtABCG14 dimerization partners by scanning the remaining *atabcg* single-gene mutants, but none of them displayed a phenotype similar to that of *atabcg14* [107].

At the same time, Zhang et al. were systematically studying the AtABCG subfamily. Similarly, to the authors of the previous study, they noticed that *atabcg14* mutants had a characteristic phenotype, which manifested as CK deficiency in shoots and an increase in CK concentration in roots. Expressing a CK signal reporter in *Arabidopsis* seedlings confirmed that in *atabcg14,* most CKs accumulated in roots. Conversely, CK concentration in *atabcg14* cotyledons remained lower than in the wild type. Feeding both wild-type and *atabcg14* seedlings with radiolabelled tZ-type CKs yielded the same results [109]. Put together, these results show that AtABCG14 contributes to the long-distance CK transport by exporting tZ-type CKs from roots to xylem. As proved in both works, the loss of its function leads to significant changes in CK distribution [107,109].

In rice, OsABCG18 is the closest ortholog to AtABCG14 and its properties and function are comparable. The *osabcg18* mutants accumulated tZ, tZR, and DHZ in roots, while their respective concentrations in shoots decreased. Experiments employing radiolabelled tZ directly demonstrated that the *osabcg18* mutation lowers root-to-shoot CK translocation. It was further shown that OsABCG18 is also involved in the export of iP, iPR, and cZR [110]. Both AtABCG14 and OsABCG18 are localized to plasma membrane [107,109,110].

Recent studies have revealed that CK membrane transport might be also mediated by two members of the “Aza-Guanine Resistant” (AZG) family—AtAZG1 and 2, previously identified as adenine and guanine importers [111,112,113].

Biochemical characterization of AtAZG1 in yeast expression system has revealed its particular affinity towards adenine (the mean *K_M_* value has been reported as 1.62 µM). In the same system, AtAZG1-mediated adenine uptake was strongly inhibited by kinetin, tZ, BAP, and to a lesser extent also by iP. Overexpression of *AtAZG1* in *Arabidopsis* seedlings enhanced their ability to accumulate adenine, although the uptake rate in seedlings bearing *atazg1* loss-of-function mutation remained similar to the wild type. Furthermore, adenine uptake mediated by the overexpressor seedlings was inhibited by tZ, similarly to the experiments in yeast. The *atazg1* mutant roots were on the other hand less sensitive to exogenously applied CKs. Another important feature of AtAZG1 is its interaction with PIN1 (“Pin-Formed”), an auxin cellular exporter. AtAZG1 was shown to co-localize with PIN1 and to stabilize it on the plasma membrane in *Arabidopsis* root cells, together with AtAZG2. Based on these results, it has been proposed that AtAZG1 modulates root cell architecture by regulating intracellular auxin:CK ratio [113].

AtAZG2 is found mainly in root primordia and is localized to plasma membrane and ER. Expression of *AtAZG2* gene is stimulated by auxins. In *AtAZG2*-expressing yeast cells, adenine uptake was strongly inhibited by iP, kinetin, BAP, and tZ. Upon ectopic expression of *AtAZG2* in *Arabidopsis* seedlings, an increase in tZ uptake was observed. However, tZ uptake in *atazg2* mutant plants was not impaired [112].

Expression of *TCSn::GFP* [106] in *Arabidopsis* has shown that CK signalling output is lowered in the proximity of *atazg2* lateral root primordia. It has been therefore proposed that AtAZG2 contributes to the inhibition of lateral root emergence by modifying CK distribution, similarly to AtPUP14 [112].

A brief summary of membrane transporters that had been studied in relation to CK transport is given in Table S1.

## 9. Cytokinin Paracrine Movement and Long-Distance Transport Open Up another Layer of Cytokinin Homeostasis Maintenance

Up to this point, CK homeostasis has been discussed in the context of isolated cells. In plant tissues, neighbouring cells are linked via symplast and apoplast. These connections enable, among other things, the paracrine hormonal signalization. Paracrine effects of CKs were studied in transgenic tobacco plants harbouring bacterial *IPT* gene under an inducible promoter [114]. Induction of this construct at a specific spot led to a local change of phenotype, suggesting CK overproduction. De Rybel et al. mention that CK biosynthesis and signalling do not necessarily occur in the same cells. For their model of auxin-CK crosstalk during vascular tissue formation, they consider paracrine movement of CKs between CK-producing xylem and CK-responsive neighbouring cambial cells [115].

At the level of whole plants, CKs are transported via vascular tissues. First pieces of evidence come from early studies focused on the distribution of radiolabelled CKs among different plant tissues and organs. Upon application of [^3^H]-tZR to pea (*Pisum sativum*) root nodules and leaves, portions of the total radioactivity (comprising both the original tracer and its metabolites) were detected in other organs after eight days. The radiolabelled CKs were more readily transported from the root nodules than from the leaves [116]. Similarly, relocation of [^14^C]-BAP applied to cocoa (*Theobroma cacao*) leaves at various stages of emergence was observed in both acropetal and basipetal direction [117].

Further information on CK long-distance transport has been obtained by means of CK profiling in vascular tissues. In xylem, the predominant CK species is tZR [67,71,72,73]. It has been also shown that the root-to-shoot translocation rate of CKs via xylem increases in plants treated with exogenous CK or with a protonophore, which impairs proton-dependent uptake of CKs by root cells [118]. Osugi et al. reported that tZ is transported from roots to shoots as well. They also suggested that both tZ and tZR may have their distinct roles as long-distance signalling molecules [119]. In phloem, both iPR and iPR monophosphate (iPRMP) are present in relatively high concentrations [67,74].

The long-distance CK transport was further examined using grafting experiments. Matsumoto-Kitano et al. focused on a quadruple *ipt* mutant, *atipt1;3;5;7*, which is strongly impaired in CK biosynthesis [38,120]. This mutant displayed a characteristic phenotype, including decreased shoot growth, increased root elongation, and decreased root perimeter (due to its inability to develop cambium), which could be recovered by applying exogenous tZ. Endogenous levels of both tZ-type and iP-type CKs were significantly lower in the mutant than in the wild type. Grafting a mutant shoot on a wild-type root restored the shoot phenotype. The level of tZ-type CKs in the shoot recovered too, while that of iP-type CKs remained decreased. An analogous observation was made after grafting a wild-type shoot on an *atipt1;3;5;7* mutant root—the root phenotype and iP-type CK contents became comparable to the wild type, but tZ-type CK contents remained as low as in the mutant plants. Altogether, these results suggest that tZ-type CKs are mostly transported from roots via xylem as a shootward signal and iP-type CKs are mostly transported from shoots via phloem as a rootward signal [67,120].

As already mentioned, CK export to xylem is mediated by AtABCG14 [107,109]. Mechanisms of CK unloading, however, remain unknown so far. In relation to the role of AtABCG14, one may assume that sink tissues express another type of CK-specific transporter. Hot candidates for such position might be the members of families such as PUP, ENT, ABC or even AZG. On the other hand, we cannot rule out that xylem CKs are gradually depleted via unloading processes independent of membrane-bound carriers.

## 10. Future Perspectives

In this review, we address CK distribution among different compartments, cells, and tissues as well as the physical and chemical facets of the underlying transport processes. In a given subcellular compartment, concentrations of various CK species and forms are regulated by both membrane transport and metabolism. These two mechanisms are not completely independent of each other—membrane transport can provide substrates for enzyme-catalysed reactions and remove reaction products, both of which affect the thermodynamic equilibrium within the compartment. Similarly, metabolic conversions regulate concentrations of substrates available for different membrane-bound carriers. So far, it seems that CK recognition by membrane-bound carriers is governed by the substrate form (i.e., the moieties attached to the free base) rather than by their side-chain character. This can be demonstrated by the differential preferences of ENTs, which recognize CKs in the form of ribosides, and PUPs, which prefer free CK bases [81,102], similarly to the newly characterized AtAZG1 and 2 [112,113]. AtPUP14 has been characterized as a tZ-specific carrier; yet, when expressed in mesophyll protoplasts and tobacco microsomes, it displayed a slightly higher affinity towards other free CK bases (kinetin, BAP, iP) and adenine than towards tZR [28]. AtABCG14 has been shown to regulate the distribution of tZ-type CKs in various forms, but its biochemical characterization has not been successful [107,109]. It follows that biochemical conversions between different CK forms, such as glycosylation, phosphorylation, etc., affect both thermodynamic and kinetic aspects of carrier-mediated membrane transport.

A question arises whether there are membrane-bound transporters recognizing CK nucleotides and glucosides or whether these are converted to other CK types prior to translocation between compartments. As pointed out by Šmehilová et al., CK glucosylation in *Arabidopsis* is mostly confined to the cytoplasm, but most CK glucosides are to be found in the apoplast. Such situation advocates for the existence of a (so far unidentified) CK glucoside exporter [76]. Similarly, presence of CK glucoside carriers on tonoplast could explain relatively high abundance of CK glucosides in *Arabidopsis* vacuoles. Furthermore, *Arabidopsis* plastids contain high amounts of CK glucosides as well as of CK nucleotides [77]. It remains to be examined whether these CKs are transported between plastids and the cytoplasm or whether they rather play a role of buffer CK pools, balancing the levels of biologically active CKs in their particular compartments.

**Figure 4:**
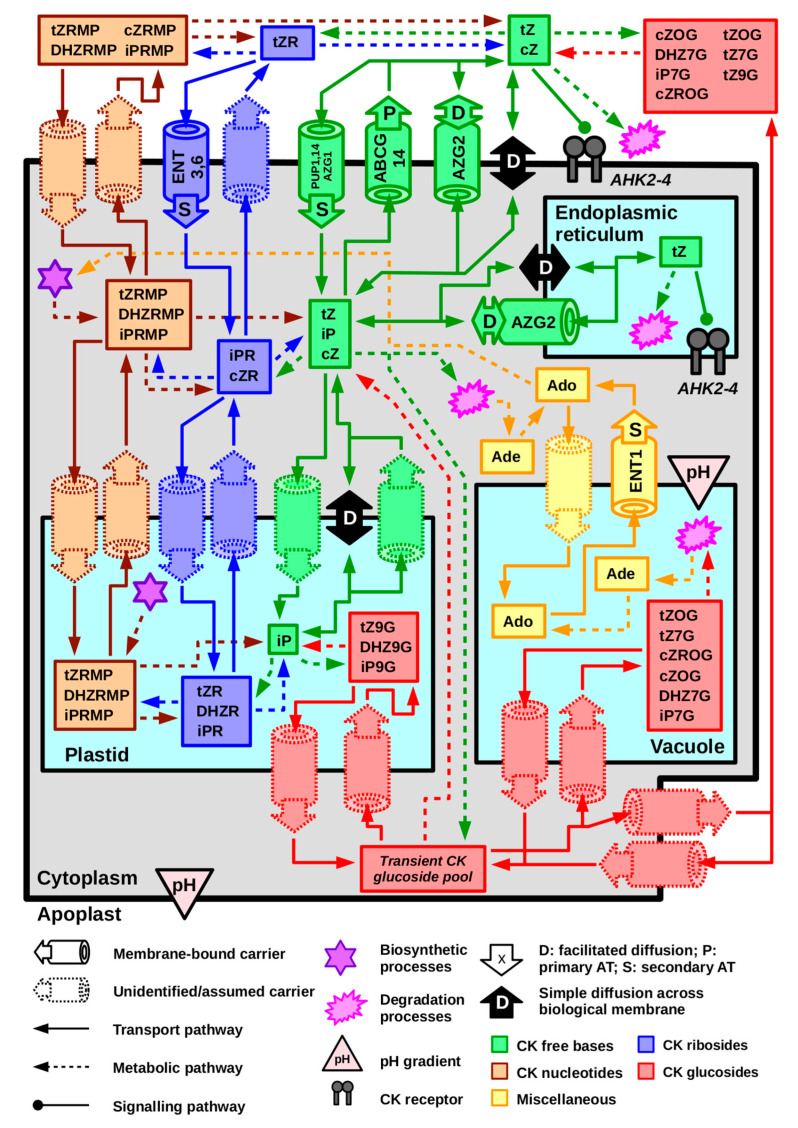
A proposition of a comprehensive scheme of cytokinin (CK) cellular homeostasis in *Arabidopsis* based on the current knowledge of cytokinin cellular trafficking in vascular plants. The plant cell is reduced to a simplified model depicting a plastid, a vacuole, endoplasmic reticulum, cytoplasm, and apoplast. Free CK bases, ribosides, nucleotides, and glucosides are grouped into their respective pools. Labels within the pools mark CK species that have been found in the given compartment in a significant amount [70,77]. Membrane-bound carriers with known subcellular localization are depicted together with a symbol indicating which kind of membrane transport process they mediate. Note that in case of free bases, which might be considered more hydrophobic than other CK species (refer to Figure 2), simple diffusion across the membrane is considered as well. Several putative membrane-bound carriers have been added to show possible transport routes that haven’t been characterized or addressed yet. However, it is not implied that all of them have to be actually involved in CK trafficking. Light pink triangles indicate pH gradient, with the point located on the more acidic side of the membrane.

In Figure 4, we present a scheme of CK distribution in *Arabidopsis* at the cellular level. Different CK forms (i.e., free bases, ribosides, nucleotides, and glucosides) are grouped into pools. Depicted flows among these pools represent both confirmed and hypothetical CK transport pathways. This summary is meant to give a general idea of all possibilities of CK cellular traffic (and to demonstrate its complexity), it is not implied that all the depicted processes do necessarily occur. At the same time, one should note that even if certain transport process occurs, it does not have to be a sign of physiological importance, but also simply a result of physicochemical conditions at the given moment.

CK-recognizing transporters are classified as CK-specific or non-specific, based on how efficiently they discriminate among structurally similar substrates. The former comprise AtPUP14 [28], AtABCG14 [107,109], and possibly AtAZG1 and 2 [112,113], and the latter the remaining PUPs and ENTs [81,101].

While the importance of specific CK transport is hardly disputable, the role of non-specific membrane-bound carriers should not be overlooked. Even if further research finds no direct link between their function and CK signalization, they can be still involved in maintaining cellular CK balance in response to changes of CK-related enzyme activities and distribution of different CK metabolites.

In the very last part of the previous section, we also address the role of non-specific mechanisms in CK long-distance transport. While not ruling out the involvement of CK-specific membrane-bound carriers (such as AtABCG14-mediated tZ-type CK export to xylem), CK translocation via vascular tissues can be co-governed by source-sink relations. Source and sink powers are results of actual CK concentrations, which can be determined by enzyme-catalysed metabolism in the respective tissues.

Concerning long-distance CK transport, it is also worth further examining the distribution of radiolabelled CK tracers. In several studies, such tracers were applied on certain parts of intact plants, and subsequently, total radioactivity was measured in target tissues [107,109,116,117]. However, exact composition of the radioactive fraction was not analysed. Knowing which metabolites of the original tracer are distributed via long-distance transport would further help to determine which processes are relevant for CK distribution among source, vascular, and sink tissues. Moreover, it would be interesting to address whether molecules entering the vasculature from the source tissue (or at least a portion of them) travel to the sink, or whether long-distance CK translocation occurs in a relay manner. The latter means that the initial CK load acts as a paracrine signal, triggering CK production in the nearby cells and further propagation of the newly synthesized CK molecules.

CK transport processes contribute to the regulation of CK signalling activity. In Figure 4, we propose a scheme representing possible flows among intracellular and apoplastic CK pools. Regarding CK signalling, the complex scheme can be simplified to apoplast and ER, where binding of CKs to their receptors occurs [19,25,26,27,29], and cytoplasm. In such model, CK signalling is up-regulated by exporting CKs from cytoplasm to apoplast or ER and down-regulated by the opposite processes. Availability of biologically active CKs in apoplast and ER is also mediated metabolically, via enzyme reactions. As discussed above, these reactions can also affect transport rates by regulating CK concentration gradient between concerned compartments.

So far, two CK transporters have been shown to regulate CK signalling output—AtPUP14 and AtAZG2 [28,112]. AtPUP14 is localized to the plasma membrane and mediates energy-dependent CK uptake to cytoplasm, preventing apoplastic CKs from triggering cellular response. Compared to AtPUP14, AtAZG2 is more versatile—it is located to both the plasma membrane and ER and mediates bidirectional facilitated diffusion. It can follow, for instance, that the combination of active AtPUP14 and inactive AtAZG2 results in CK accumulation in cytoplasm and down-regulation of all CK receptors [112].

Considering the scheme in Figure 4, it is apparent that a large number of hypothetical pathways such as the one mentioned above could be designed. However, it is not implied that every CK movement has to ultimately result in the change of CK signallization output. As discussed throughout this paper, CK transport can also occur via processes driven by chemical properties of CK molecules and their transient concentration gradients. When discussing links between CK transport and signalling, it is therefore necessary to look for direct evidence and to keep in mind that correlation does not prove causality.

## Supplementary Materials

The following is available online at https://www.mdpi.com/article/10.3390/ijms22073428/s1, Table S1: A summary of membrane-bound transport proteins which have been examined regarding the CK transport.
