# Supplementary material for: Differential Subcellular Distribution of Cytokinins: How Does Membrane Transport Fit into the Big Picture?
Source: Int J Mol Sci. 2021 Mar 26;22(7):3428. doi: 10.3390/ijms22073428 (PMC8037549; doi:10.3390/ijms22073428)
Supplement: Supplementary file 1 [file ijms-22-03428-s001.pdf]

Table S1. A summary of membrane-bound transport proteins which have been examined regarding the CK transport. For each transporter, a brief overview of its involvement in the CK transport (and possibly other functions) is provided. If available, measured K_M values for CKs are given as well. Respective transport mechanisms, expression patterns, and subcellular localizations are listed in the following columns. SAT: secondary active transport; PAT: primary active transport; *At*: *Arabidopsis thaliana* (mouse-ear cress); *Os*: *Oryza sativa* (rice); *Nt*: *Nicotiana tabacum* (tobacco); BAP: 6-benzylaminopurine, cZ: *cis*-zeatin, DHZ: dihydrozeatin, iP: isopentenyl adenine, iPR: isopentenyl adenine riboside, tZ: *trans*-zeatin, tZR: *trans*-zeatin riboside; *N/A*: not available.

Name	*Relation to CK Transport*	*Other Notable Properties*	*Transport Mechanism*	*Expression Pattern*	*Subcellular Localization*	*References*
AtPUP1	Adenine uptake was competitively inhibited by kinetin and tZ. Direct tZ uptake was observed; it was inhibited by kinetin and iP (both in yeast). Measured K_M values (μM) – **kinetin:** 20, **tZ:** 35 (both in yeast).	Uptake of adenine, cytosine, hypoxanthine, nicotine, and caffeine was observed (in yeast). Involved in uptake of pyridoxine (in *Arabidopsis* protoplasts).	Proton-coupled SAT	Flowers, leaves, petioles, siliques, stems, veins.	*Plasma membrane.*	[60,61,103]
AtPUP2	Adenine uptake was inhibited by iP, kinetin, BAP, tZ, and cZ (in yeast).	The adenine uptake rate was lower than that mediated by AtPUP1 (in yeast).	Proton-coupled SAT	Flowers, leaves, petioles, roots, veins.	*N/A*	[61]
AtPUP3	*N/A*	No transport activity observed.	*N/A*	Flowers.	*N/A*	[61]
AtPUP14	Ectopic expression alters CK response pattern. Direct of tZ was observed in mesophyll protoplasts and tobacco microsomes. This uptake was inhibited by iP, BAP, and adenine.	Abundant expression in whole plants throughout various developmental stages.	ATP-dependent transport	Flowers, roots, rosettes, seeds, stems.	*Plasma membrane.*	[28,100]
AtENT1	Mutation **did not** alter iPR nor tZR uptake by *Arabidopsis* hypocotyl explants.	Uptake of adenosine, guanosine, inosine, and cytidine was observed (in yeast). Involved in adenosine export from the vacuole to the cytoplasm.	Proton-coupled SAT	Flowers, leaves, siliques, stems, roots.	*Plasma membrane, tonoplast.*	[62,91–93,95]
AtENT3 (FUR1)	Mutation led to a decrease in iPR and tZR uptake by *Arabidopsis* hypocotyl explants. No significant inhibition of adenosine uptake was observed (in yeast).	Uptake of adenosine, guanosine, inosine, cytidine and uridine was observed (in yeast).	Proton-coupled SAT	Flowers, leaves, siliques, roots.	*Plasma membrane.*	[62,67,95]
AtENT6	Mutation led to a decrease in iPR and tZR uptake by Arabidopsis hypocotyl explants. Adenosine uptake inhibited by iPR and tZR (in yeast). Measured K_M values (μM) – **iPR:** 17, **tZR:** 630 (both in yeast).	Uptake of adenosine, guanosine, inosine, cytidine and uridine was observed (in yeast).	Proton-coupled SAT	Flowers, leaves, siliques, stems, roots.	*Plasma membrane.*	[67,88,95]
AtENT7	Mutation led to a decrease in iPR and tZR uptake by *Arabidopsis* hypocotyl explants. No significant inhibition of adenosine uptake was observed (in yeast).	Uptake of adenosine, guanosine, inosine, cytidine and uridine was observed (in yeast).	Facilitated diffusion	Flowers, leaves.	*N/A*	[67,95]
AtENT8 (SOI33)	Mutation led to a decrease in iPR and tZR uptake by Arabidopsis hypocotyl explants. Mutants were also less sensitive to exogenous CKs, compared to the wild type. Conversely, over-expressing plants were more sensitive to them.	N/A	Proton-coupled SAT	Flowers, leaves, roots, siliques, stems.	*N/A*	[62,95]

AtABCG14	Loss-of-fucntion mutants were unable to translocate tZ-type CKs from roots to shoots. Mutants were impaired in growth and lateral root emergence. Application of exogenous tZ and grafting mutant shoots on wild-type roots (but not vice versa) restored the wild-type phenotype. Transport activity in transgenic yeast cells was not observed.	Forms strict heterodimers with AtABCG11 in co-immunoprecipitation assays.	PAT	Mainly in roots.	*Plasma membrane.*	[107,109]
AtAZG1	Adenine uptake was inhibited by iP, kinetin, BAP and tZ (in yeast). tZ uptake was observed both in yeast and *Arabidopsis* seedlings. Overexpression led to uptake increase, while loss-of-function mutation led to decreased concentrations of tZ-type CKs after tZ treatment (both in *Arabidopsis* plants).	Both AtAZG1 and 2 stabilize PIN1, an auxin transporter, on plasma membrane.	Proton-coupled SAT	Cotyledons, flowers, leaves (rosette and cauline), stems, roots.	*Plasma membrane.*	[113]
AtAZG2	Adenine uptake was inhibited by iP, kinetin, BAP, and tZ (in yeast). Ectopic expression in Arabidopsis led to an enhanced uptake rate of tZ, while in loss-of-function mutants, the tZ uptake rate remained similar to that in the wild type. The mutants were less responsive to CK signalling, though. Measured KM values (nM) – tZ: 810 (in *Arabidopsis* cell suspension).	The loss-of-function mutation enhances the lateral root density in presence of nitrate.	Facilitated diffusion	Flowers, roots.	*Endoplasmatic reticulum, plasma membrane.*	[112]
OsPUP1	Overexpression in rice plants alters CK concentrations in shoots, roots, and panicles. Root-to-shoot translocation of CKS is impaired. Endogenous CK application to roots triggered less pronounced response in shoots.	OsPUP1 and 4 act antagonistically in several aspects. When simultaneously overexpressed, OsPUP4 overcame some phenotype traits typical for OsPUP1.	N/A	Leave blades and sheats, mature roots, panicles, stems.	*Endoplasmatic reticulum.*	[105]
OsPUP4	Overexpression in rice plants alters CK concentrations in shoots, roots, and panicles. Shoot-to-root translocation of CKs is impaired. Endogenous CK application to shoots triggered less pronounced response in roots.		N/A	Leave blades and sheats, mature roots, panicles, stems.	*Plasma membrane.*	[104,105]
OsPUP7	Higher amounts of iP and iPR were detected in mutant plants. The amounts of tZ-like CKs did not change. Mutant plants displayed phenotype suggesting CK transport impairment.	Ability to complement mutant yeast deficient in caffeine uptake.	N/A	Grains, leaves, pistil, spikelets, stems.	*Endoplasmatic reticulum.*	[68,104]
OsENT2	Adenosine uptake was inhibited by iPR, but not by tZR. Direct uptake of both iPR and tZR was observed (all in yeast). Measured KM values (μM) – iPR: 32, tZR: 660 (both in yeast).	Uptake of adenosine, guanosine, inosine, cytidine and uridine was observed (in yeast).	Proton-coupled SAT	Grains, leaves, roots, stems, veins.	*N/A*	[66]
OsABCG18	The loss-of-function mutants are impaired in their ability to translocate tZ, tZR, and DHZ from roots to shoots. When expressed in tobacco leave explants, iP, iPR, and cZR we recognized as substrates too.	The closest ortholog of AtABCG14 in rice.	Primary active transport.	Leaves, panicles, roots, stems.	*Plasma membrane.*	[110]

NtPUP1	A 10-fold excess of unlabelled kinetin did not significantly inhibited uptake of [14]-C nicotine (in yeast).	A nicotine-specific transporter; more than 50% homology with AtPUP1.	N/A	Mainly root tips.	*Plasma membrane.*	[99]
